# Progress in Fertility Preservation Strategies in Turner Syndrome

**DOI:** 10.3389/fmed.2020.00003

**Published:** 2020-01-24

**Authors:** Mudan Ye, John Yeh, Ioanna Kosteria, Li Li

**Affiliations:** ^1^Department of Gynecology and Obstetrics, Guangzhou Women and Children's Medical Center, Guangzhou Medical University, Guangzhou, China; ^2^Department of Gynecology, Obstetrics and Reproductive Biology, Harvard Medical School, Boston, MA, United States; ^3^Division of Endocrinology, Metabolism and Diabetes, First Department of Pediatrics, Medical School, National and Kapodistrian University of Athens, Agia Sophia Children's Hospital, Athens, Greece

**Keywords:** Turner syndrome, fertility preservation, cryopreservation of oocytes, cryopreservation of ovarian tissues, cryopreservation of embryos

## Abstract

Growth retardation and gonadal dysgenesis are two of the most important clinical manifestations of Turner syndrome (TS). As premature ovarian failure generally occurs early in life in women with TS, these patients should be counseled and evaluated as early as possible for discussion of optimal and individualized fertility preservation strategies. Infertility seriously affects the quality of life of women with TS. For those who have ovarian reserve, the theoretical options for future fertility in TS patients include cryopreservation of oocytes, ovarian tissues, and embryos. For those who have already lost their ovarian reserve, oocyte or embryo donation, gestational surrogacy, and adoption are strategies that allow fulfillment of desire for parenting. This review describes the etiologies of infertility and reviews the fertility preservation strategies for women with TS.

## Introduction

Turner syndrome (TS) is one of the most common sex chromosome abnormality in women. The incidence rate of TS in female newborns is about 1/2,500 (1). The karyotypes of TS are 45,X monosomy accounting for about 50% of the cases, 45X/46XX mosaicism accounting for about 20–30%, and the rest is other X chromosome structural abnormalities (2). It is usually characterized by hypergonadotrophic hypogonadism due to gonadal dysgenesis leading to premature ovarian failure with subsequent infertility. About 80% of women with TS do not have spontaneous puberty, and the ovarian reserve in 90% of women with TS will be depleted before adulthood (3). Fertility preservation in women with TS needs to be offered in a timely fashion. In this paper we review available methods for the detection of ovarian reserve, as well as the different for fertility preservation in women with TS.

## Fertility Reserve and Ovarian Reserve Testing in Women With TS

### Ovarian Reserve

Ovarian reserve refers to the number of primordial follicles contained in the human ovarian cortex. The formation of the ovarian reserve in females with normal chromosomal karyotype begins at the fetal stage. At that stage, 100–2,000 primitive germ cells enter the genital ridge and proliferate in large numbers, reaching the maximum amount of germ cells in the second trimester of pregnancy. After that, the number of germ cells gradually decreases, and no new germ cells are formed. Mamsen et al. (4) observed that the number of germ cells in 53 fetal ovaries at different stages of pregnancy increased from an average of 7,200 at 7 weeks of gestation to 4,933,000 at 19 weeks of gestation, but about 85% of the germ cells were lost before birth and the number dropped to about 600,000 at birth. The number of oocytes decreases with age, regardless of whether a woman has an ovulatory cycle or not. By the age of 37, the number of eggs is ~25,000, and the rate of reduction will be accelerated. By the age of 51, the number of remaining eggs will be <1,000 in the average woman (5).

Studies have shown that the ovarian development in fetuses with TS is normal before 12 weeks of gestation, but oocyte loss is accelerated in many of them after 18 weeks of gestation. In normal karyotype fetuses, oocyte division begins at 18 weeks of gestation, primordial follicles are observed at 20 weeks of gestation, and antral follicles are observed at 26 weeks of gestation. On the contrary, at the same gestational week, oocytes are present in the ovaries of 45,X fetuses, but no follicles are observed. Finally, the number of germ cells at birth is significantly less than that of females with normal karyotypes at the same developmental age (6, 7). Hook et al. (8) also observed that, in the early stage of a fetus with abnormal chromosomal karyotype, there were normal numbers of germ cells in the reproductive ridge. However, in the second trimester of pregnancy, the number of germ cells was significantly lower than that of the normal fetus, indicating that the rate of apoptosis of germ cells during development was higher in the fetus with abnormal chromosomal karyotype. Modi et al. (9) made a semi-quantitative analysis of the ovaries of 16 normal fetuses and 4 TS fetuses at 15–20 weeks of gestation, showing that TUNEL (terminal deoxynucleotidyl transferase-mediated deoxiuridinetriphosphate nick-end labeling) -positive cells were present in 3–7% of normal fetuses and 50–70% in fetuses with TS, also suggesting that the rate of apoptosis of reproductive cells in fetuses with TS was higher than that of normal fetuses. Therefore, it was speculated that the accelerated apoptosis of germ cells in women with TS in the intrauterine growth period may be the main mechanism of follicular depletion in this syndrome.

### Chromosome Karyotype and Ovarian Reserve in TS

The chromosome karyotype of women with TS has been correlated with the size of ovarian primordial follicular pool and spontaneous puberty. Hankus et al. (10) compared the karyotypes of 110 patients aged 10.7 ± 4.0 years, and the results showed that in 48% patients with spontaneous puberty, most of them were non-45,X girls. Mamsen et al. (11) evaluated follicular density, morphology, and health in the ovarian cortex of 15 patients aged 5–22 years with TS, and found that 9 women with TS had follicles with chimeric karyotype. Bernard et al. (12) found that 27 patients among 480 women with TS (5.6%) had a total of 52 spontaneous pregnancies. The two predictive factors which correlated with the occurrence of spontaneous pregnancy were spontaneous menarche and mosaic karyotype. However, Mortensen et al. (13) reported that a small number of women with TS with karyotype 45,X can have spontaneous puberty and regular menstruation, and even spontaneous pregnancy. Therefore, it is difficult to accurately predict ovarian reserve and fertility potential in women with TS on the basis of chromosomal karyotype alone.

In addition, studies have shown that the chromosomal structure of women with TS has an impact on ovarian function. Hreinsson et al. (3) studied and carried out histological analysis on the ovaries of 9 women with TS, among which 8 had follicles. Their results showed that there were more follicles in the ovaries of patients with mosaicism and younger women with TS. Quilter et al. (14) reported that in a comparative genomic hybridization (aCGH) analysis of 42 patients with idiopathic POF (Premature ovarian failure), 15 were found to have copy number variations (CNV) on chromosome X and chromosome 7. Mercer et al. (15) conducted a more in-depth study on the fertility of 20 patients with long arm variations of the X chromosome, showing that loss of Xq26-Xq28 has the greatest impact on ovarian function. These studies suggest that abnormal X chromosome structure and even CNV on autosomes may be associated with the fertility of women with TS.

### Evaluation for Potential Fertility in Women With TS

As the ovarian reserve capacity varies among women with TS, it is important to select these patients who can preserve their fertility as early as possible. Anti-Mullerian Hormone (AMH) is a reliable marker of follicular reserve in normal adults. Even though there was a study showing that AMH is suitable for use in young or adolescent women, there is few data available on AMH in childhood (16, 17). A retrospective study of 28 women with TS with different karyotypes was conducted by Lau et al. (18) to compare their development and menstruation, and to measure serum Follicle-Stimulating Hormone (FSH) levels in order to assess their fertility. The results showed that patients were more suitable for fertility preservation when they had spontaneous menarche, had at least one normal ovary confirmed by ultrasound, and their serum FSH levels were below 40 IU/L. Birgit et al. (19) reported on laparoscopic examination and ovarian biopsy in 57 girls with TS aged 8–19.5 years. Ovarian cortical tissues were obtained from 47 of these TS girls with non-streak ovaries for follicular analysis and cryopreservation. Luteinizing Hormone (LH) and FSH levels were measured in 30 of them, and AMH levels were measured in 43 of them. Follicles were detected in the ovaries of 26% of these women, and the number of follicles varied based on the levels of LH, FSH, and AMH, karyotype, spontaneous puberty and menarche.

Based on the above studies, it appears that women with TS may benefit by having an assessment of ovarian reserve at the age of 13 or 14, so as to consider their options for fertility preservation in the most timely manner. The diagnostic parameters are mainly serum AMH level, serum FSH level, karyotype analysis, spontaneous puberty development, ovarian volume detected by ultrasound examination and follicle count. Hagen et al. and Kelsey et al. research shows the serum AMH decreased earlier than FSH, inhibin B, and E2 in prepubertal girls (16, 20). Among them, serum FSH levels, ovarian volume measured by ultrasound examination and follicle count have strong periodicity, and are interdependable, whereas AMH is a relatively stable hormone index (21). Recently, studies have also shown that there is no periodic fluctuation in serum AMH levels, and that it is better than FSH, E2, INH-B, and AFC in reflecting the downward trend of ovarian reserve function with age (22).

## Strategies for Reproductive Counseling for Women With TS

### Oocyte Cryopreservation

Cryopreservation of oocytes is an established method that may be used for both mature and immature oocytes. The first live-born baby following this technique was born in 1986 (23). After decades of development, oocyte cryopreservation has made great progress, especially with the emergence of vitrification. The pregnancy rate and live birth rate of thawed oocytes transplanted after vitrified cryopreservation were similar to those of transplanted fresh oocytes (24).

As already mentioned, in most women with TS ovarian reserve may be depleted before or relatively soon after puberty. Moreover, women with TS may have oocytes with normal or abnormal karyotypes depending on their own karyotypes. Oocyte cryopreservation may provide a chance for selecting the normal karyotype oocytes resulting thus in an embryo with a normal karyotype. Therefore, offering cryopreservation with oocyte vitrification in patients in women with TS before ovarian reserve is a highly considerable option. Due to chromosomal abnormalities in women with TS, not all oocytes develop with a normal karyotype and resulting fertilized embryo's also carrying the chromosomal abnormality, with females developing TS and males being carriers of the condition. Fortunately, pre-implantation genetic screening (PGS) technology is mature and can be used to screen stored oocytes and embryos for chromosomal abnormalities. Given the technological advances in oocyte and embryo screening, it can be predicted that women with TS are expected to successfully conceive and produce healthy babies through these techniques.

#### Cryopreservation of Mature Oocytes

**Figure 1:**
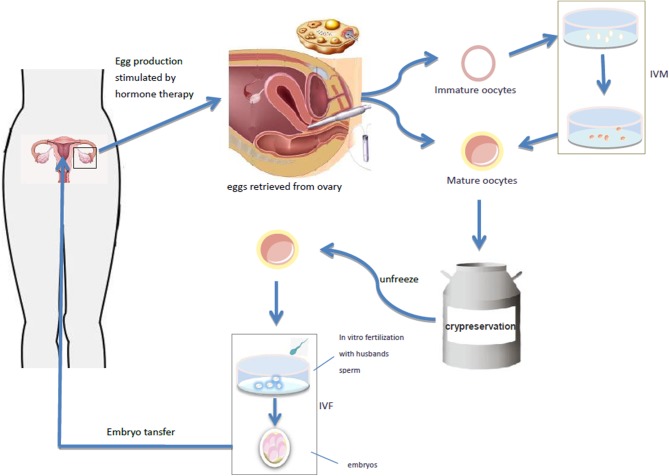
The follicular maturation is monitored by ultrasound, and then the oocyte is collected by ultrasound guided ovum retrieval. The collected oocytes are vitrified and cryopreserved, and the frozen oocytes will be thawed and fertilized at the appropriate future time. It takes out the immature oocytes from the ovary and puts them into the culture medium simulating the follicular microenvironment *in vivo*, and cultures the oocytes *in vitro* to mature stage, and then fertilization and pregnancy occurs through IVF-ET. Oocyte maturation can be directly performed by IVF-ET and pregnancy.

Mature oocytes mainly refer to the oocytes in the MII (meiosis II) stage. In natural cycles, only 1–2 oocytes at one time can reach full maturity at MII stage, and the rest will degenerate after atresia. Ovarian stimulation by exogenous gonadotropin administration to promote ovulation is the best approach to collect multiple mature oocytes in a relatively short period of time (25, 26). The follicular maturation is monitored by ultrasound, and then the oocyte is collected by ultrasound guided ovum retrieval. The collected oocytes are vitrified and cryopreserved, and the frozen oocytes will be thawed and fertilized at the appropriate future time (Figure 1). So far, this technique has been successfully used in a small number of women with TS (18, 25, 27, 28). Unfortunately, the number of mature oocytes retrieved in a single cycle is very small in women with TS. Therefore, the procedure often has to be repeated in order to finally obtain more than 10 mature oocytes. Then, it is difficult for a young patient (adulthood).

#### Cryopreservation and *in vitro* Maturation of Immature Oocytes

Immature oocytes refer to the oocytes at GV (germinal vesicle) or MI (meiosis I) stage. The collection of immature oocytes does not require gonadotropin stimulation. They may be directly harvested from removed ovarian tissue and their size usually ranges from 2 to 8 mm. *In vitro* maturation (IVM) of immature oocytes is also an assisted reproductive technology, which takes out the immature oocytes from the ovary, transfers them into appropriate culture medium simulating the follicular microenvironment *in vivo*, cultures the oocytes *in vitro* to mature stage, and then fertilization and pregnancy may occur through IVF-ET (29) (Figure 1). IVM can completely bypass ovulation promoting process, simplify IVF program and shorten treatment time, offering thus significant advantages.

Oocyte cryopreservation techniques use autologous oocytes and do not require sperm fertilization, thus avoiding religious and ethical issues. If a patient is unable to gestate because of pregnancy contraindications, as in a number of selected cases of TS, these techniques may also provide the possibility of retaining oocytes to achieve pregnancy via surrogacy. However, it should be noted that for women who cannot tolerate ovarian stimulation and for some TS girls who either do not have a partner or are at a very young, even prepubertal age, transvaginal oocyte retrieval may not be easily accepted by them or-in the latter case-by their parents, and may not be applicable to them. Transabdominal oocyte retrieval is not generally recommended, and in this case, a more appropriate alternative approach should be offered. Moreover, not all oocytes obtained are suitable for fertilization, and some oocytes may have abnormal karyotypes. Therefore, chromosome screening should be routinely carried out before embryo implantation in order to screen out aneuploidy embryos and to improve the outcome of pregnancy. However, this technology is currently being evaluated and has never been studied in women with TS.

### Ovarian Tissue Cryopreservation and Ovarian Transplantation

Fertility preservation techniques for cryopreserved ovarian tissues are still in the experimental stage, but it has been reported that cryopreserved ovarian tissues after orthotopic transplantation can restore ovarian function and lead to spontaneous pregnancy. Donnez et al. (30) first reported in 2004 that a patient with premature ovarian failure caused by chemotherapy for Hodgkin's lymphoma had a successful pregnancy and delivered a baby after orthotopic transplantation of her cryopreserved ovarian tissue. According to relevant data, by 2017 the number of live births through this technology has exceeded 130 babies, with live birth rate constantly increasing. As the ovarian tissue is preserved in multiple pieces, the transplantation process can and may be repeated, providing each time a new opportunity to restore ovarian function. There have been reports that ovarian function can be restored for 4–5, or even 7 years. This technology can not only help preserve the fertility of patients, but also help restore endocrine function (31–35). However, so far, there have been no reports in women with TS. Ovarian tissue transplantation is the most appropriate option for prepubertal girls with TS and merits further investigation.

#### Cryopreservation of Ovarian Tissue

This technique requires laparoscopic surgery to obtain the ovarian cortex. Because the ovarian reserve of women with TS is low and the number of follicles in the ovary is small, to improve the success rate, it is recommended to obtain as much ovarian cortex as possible, sometimes even part or the whole ovary. Ovarian cortex containing follicles was cryopreserved. The cryopreserved ovarian tissue is thawed when women with TS are ready for pregnancy. The location of ovarian transplantation depends on whether the patient retains part of the ovary when the ovarian cortex is removed. If the ovary is retained, it can be orthotopically transplanted to the ovarian remnant or mesovarium; if no ovary exists, it can be transplanted to the site where the pelvic vessels are more abundant. Mamsen et al. (11) recently reported a total of 15 women with TS aged 5.0–22.4 years old who underwent ovarian tissue cryopreservation. A substantial number of follicles was only present in girls with mosaic TS.

As the primum movens of ovarian failure in women with TS is an abnormal apoptotic cycle. The success of cryopreservation and transplantation of ovaries depends on the number of oocytes in the transplanted tissues. As the number of primitive follicles in the ovaries of TS girls is relatively small, and some oocytes may be damaged during cryopreservation, the success rate of cryopreservation in women with TS may be relatively low. In addition, the increased apoptotic cycle will resume once the grafts begin their activity, quickly diminishing the available oocytes. To improve the results, one major need is to improve graft revascularization. Some studies showed that stimulation by angiogenic and anti-apoptotic factors, such as encapsulated vascular endothelial growth factor (VEGF) or stromal cells enriched in CD34 cells before transplantation may substantially contribute to angiogenesis (31, 36–38). In conclusion, ovarian tissue cryopreservation may be applicable to women with TS who have sufficient ovarian reserve but are not sufficiently mature to cryopreserve oocytes. So far there are no reported cases of women with TS achieving successful pregnancy through this technique.

#### Ovarian Activation *in vitro*

**Figure 2:**
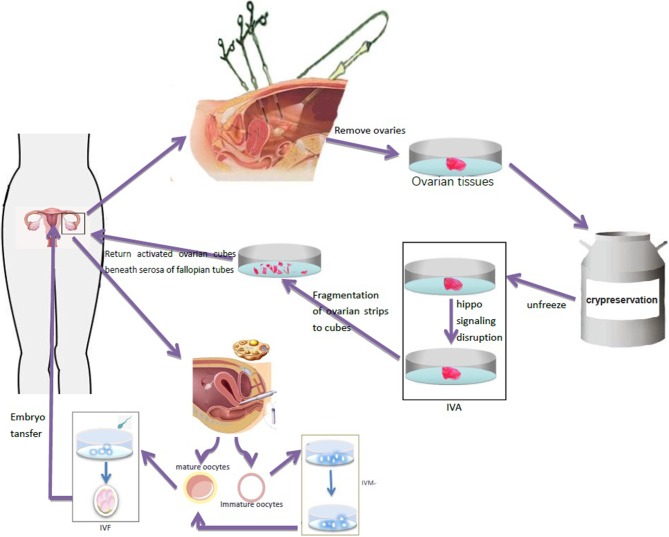
Ovarian tissue was removed by laparoscopy and frozen for preservation. After thawing, ovary fragmentation and autologous transplantation after Akt stimulation were performed to promote the growth of follicles and generate oocytes in TS patients. Immature oocytes were cultured *in vitro* to mature oocytes, which were used for IVF-ET and pregnancy.

*In vitro* ovarian activation (IVA) is a technique in which signal pathway activators are used to process ovarian tissues *in vitro* to activate primitive follicles in the ovary. Kawamura et al. (39) showed that ovarian fragments may interfere with the ovarian Hippo signaling pathway and lead to the growth of ovarian follicles. Based on the destruction of Hippo signal and AKT stimulation, primitive follicles were activated before ovarian tissue transplantation to promote the growth of follicles, restore mature oocytes, and achieve pregnancy by IVF-ET. The activated ovarian tissue can then be transplanted into the patient to increase the success rate of the ovarian transplantation (Figure 2). The first IVA baby in the world was born in Japan in 2013 (40). So far, only two cases of successful pregnancy using this technique have been reported, but none in TS, and it is still in an experimental stage (41). It can be hypothesized that it can be successfully used for women with TS with ovarian tissue transplantation and help improve their pregnancy success rate.

Studies have shown that taking multiple biopsy specimens from one ovary does not affect future hormone production, but a single ovary removal by ovariectomy can expedite menopause by 1–2 years (42). Theoretically, it may also accelerate the onset of premature ovarian failure in women with TS. Nevertheless, this technology also offers hope for fertility preservation in women with TS. Cryopreservation of ovarian tissue may be the only way to achieve fertility in some women with TS, whose ovaries cannot wait until puberty for oocyte cryopreservation, and it can even be performed in young children with TS.

### Oocyte Donation and Embryo Cryopreservation

In most women with TS, ovarian function begins to decline before puberty, and by the time many of the patients desire to get pregnant, ovaries are failing, though follicles may still exist. There is little hope for pregnancy, through ovarian stimulation and *in vitro* fertilization and all above mentioned techniques are still experimental. At that point, the best option to achieve pregnancy is by oocyte donation. Deligeoroglou et al. (43) reported three out of four patients diagnosed with TS who underwent of *In Vitro* Fertilization (ICSI-IVF) with donor oocytes and brought pregnancy to completion. They found that women with TS could get the possibility to get pregnant only when diagnosed in childhood or adolescence and undergoing hormone replacement therapy (HRT). Pregnancy rates using assisted reproduction techniques (ART) and -primarily- with donated oocytes are better in women with TS who have mature secondary sexual characteristics and almost normal size uterus. In a case report of 2008, a mother donated her oocytes to her own TS daughter and had them cryopreserved for future use (44). Letur (45) reported that 18% of women with TS benefited from this technology in 2007. Embryo cryopreservation technology is one of the most common cryopreservation techniques in assisted reproductive technology. Most women with TS have reportedly used donor oocytes for *in vitro* fertilization (IVF-DO) and successful pregnancy, with a success rate of ~37.8% (46). Andre et al. (47) reported that from 2012 to 2016, 73 women with TS obtained oocytes from 10 oocytes donation centers in France, resulting in 39 successful pregnancies after embryo transfer. However, IVF-DO has not been implemented in many countries due to ethical disputes and legal issues. For example, it is allowed by law in United States, Argentina, Russia, etc., but there is currently no relevant legal support in China.

## Pregnancy in Turner Syndrome and Complications of Pregnancy

For women with TS, there are more maternal and fetal complications irrespective of the method of conception. In 2019, Andre et al. (47) reported 151 cases of embryo transfer and 39 cases of gestation in 73 TS patients, including 24 cases of healthy delivery, 11 cases of spontaneous abortion, 3 cases of artificial abortion, 1 case of ectopic pregnancy, 1 case of non-cardiovascular death due to gestational hypertension. Moreover, the risk of spontaneous abortion is higher, mainly due to genetic abnormalities of the fetus. Intrauterine growth retardation, low birth weight, premature delivery, and stillbirths, possibly due to poor uterine environment may also occur. Women with TS who plan to pursue pregnancy need comprehensive screening and counseling before conception, including cardiac function assessment, blood pressure monitoring, echocardiography, chest MRI, abdominal ultrasound, etc. to fully understand and assess the pregnancy risk. The two most important points are prenatal genetic screening to avoid embryo chromosomal abnormalities, and pre-pregnancy cardiac assessment. Given that the cardiac output of pregnant women is about 50% higher than that of non-pregnant women and 23–50% of women with TS have congenital heart disease, the most common type being the two-leaf aortic valve (48). Because of contraction and dilatation of the aorta, pregnancy significantly increases the risk of cardiovascular complications associated with TS. Pregnancies in women with TS are at higher risk of maternal death from aortic dissection or dissection rupture or tear at the aortic root and are also at high risk of serious hypertensive disorders regardless of the use of autologous or donated oocytes (49). Hadnott et al. report, the incidence of aortic dissection in women with TS after pregnancy can be as high as 2.0%~4.8 and the risk of developing preeclampsia is about 21% (20, 50). Therefore, pre-pregnancy cardiac assessment with measurement of the aortic size index (ASI) is indispensable for women with TS who are considering pregnancy. An ASI index of >2 cm/m^2^ is considered a contraindication to pregnancy (51). In addition, because of the cephalopelvic disproportion in TS women, the cesarean section rate is higher, followed by the increased risks associated with cesarean section. In short, all women with TS who intend to carry a pregnancy should be informed of these possible risks in advance, should be evaluated before pregnancy and be closely monitored regularly during pregnancy and postpartum. Furthermore, considering the risk of pregnancy in women with TS, it is recommended to transfer only one embryo, should IVF-ET be performed, in order to minimize the risk of multiple pregnancy (49).

## Surrogacy and Adoption

Gestational surrogacy refers to the procedure by which accepts to become pregnant and give birth to a child not biologically related to her for an intended woman/couple. The source of oocytes can be autologous or donated. Because of the high risks of serious cardiovascular disease and other complications during pregnancy in women with TS, gestational surrogacy is a reasonable alternative to pregnancy in the few countries where surrogacy is legal. However, gestational surrogacy is not allowed due to lack of relevant laws in many countries, e.g., many Asian countries, especially China, but it is allowed in the United States, Russia, Ukraine, and so on. Adoption is feasible for all women with TS, and this method, as well, completely bypasses the risks of the major complications associated with a TS pregnancy.

## Conclusions

In summary, the risks of premature ovarian failure and infertility in TS are extremely high, as the ovarian reserve in girls with TS will be already exhausted before adulthood. In order to maximize the benefits of fertility preservation, it is recommended that all women with TS should be diagnosed as early as possible, evaluated for ovarian reserve, and be offered options for fertility preservation in case of residual ovarian function. Cryopreservation of oocytes and embryos are two well-established methods of fertility preservation available for women with TS. Cryopreservation of ovarian tissue is still in experimental stage, but appears to be a promising technique, especially if accompanied with the ovarian activation *in vitro* technique. For those women with TS who have lost their ovarian reserve, oocyte or embryo donation and adoption can be a way to fulfill their childcare aspirations. The risks of spontaneous abortion, fetal abnormalities, maternal complications, and mortality in women with TS are much higher than those in women with normal karyotypes. Patients with TS with pregnancy contraindications can use their own or donors' oocytes or embryos for gestational surrogacy.

## Author Contributions

MY wrote the article and searched the literature. JY and IK reviewed the literature and contributed to the writing of the article. LL contributed to the conceiving and final editing of the article.

### Conflict of Interest

The authors declare that the research was conducted in the absence of any commercial or financial relationships that could be construed as a potential conflict of interest.
